# Impact of Climate Change Education on Pregnant Women's Anxiety and Awareness

**DOI:** 10.1111/phn.13455

**Published:** 2024-10-10

**Authors:** Leyla Kaya, Esra Keles, Kürşad Nuri Baydili, Zahide Kaya, Pınar Kumru

**Affiliations:** ^1^ Department of Midwifery, Faculty of Health Sciences University of Health Sciences Istanbul Turkey; ^2^ Department of Gynecologic Oncology Kartal Lütfi Kırdar City Hospital University of Health Sciences Istanbul Turkey; ^3^ Department of Public Health, Hamidiye Faculty of Medicine University of Health Sciences Istanbul Turkey; ^4^ Department of Biostatistics, Hamidiye Faculty of Medicine University of Health Sciences Istanbul Turkey; ^5^ Department of Internal Medicine Uskudar State Hospital Istanbul Turkey; ^6^ Department of Obstetrics and Gynecology Zeynep Kamil Women and Children's Disease Training and Research Hospital University of Health Sciences Turkey Istanbul Turkey

**Keywords:** climate change anxiety, climate change, maternal health, mental health, pregnancy

## Abstract

**Objective:**

To evaluate the impact of climate change education on pregnant women's climate change awareness and anxiety.

**Study Design:**

Quasi‐experimental research with pre‐ and post‐test design.

**Methods:**

This study was conducted among pregnant women who visited a tertiary maternity hospital between April and June 2023 to assess climate change awareness, perception, knowledge, behavioral and policy expectations, and anxiety before and after the introduction of climate change education. The first phase of the study was conducted by distributing a set of questions related to sociodemographics and completing the Climate Change Awareness Scale and the Climate Change Worry Scale, followed by climate change education where pregnant women were exposed to a brochure entitled “Pregnancy and Climate Change”. After the intervention, pregnant women were assessed using the same questionnaire.

**Results:**

There was a significant decrease in pregnant women's anxiety regarding climate change (*p* < 0.001). Participants' awareness (*p* < 0.001), perception (*p* < 0.001), knowledge (*p* < 0.001), and policy expectations regarding climate change significantly increased (*p* < 0.001), while their anxiety levels decreased (*p* < 0.001).

**Conclusion:**

The study suggests that climate change education may reduce climate change anxiety among pregnant women while also enhancing their awareness, and improving their perceptions, knowledge, behaviors, and policy expectations about climate change.

## 1 Background

Climate change has become a global health threat, leading to various environmental issues, such as global warming, precipitation, fires, storms, vector‐borne diseases, and extreme weather events. These environmental problems have been linked to certain health risks to pregnant women and their fetuses, including anemia, eclampsia, low birth weight, preterm birth, and miscarriage (World Health Organization 2023). According to the World Health Organization, approximately 3.6 billion people live in areas highly susceptible to climate change. By 2030, it is estimated that climate change will cause an additional 250,000 deaths per year from malnutrition, malaria, diarrhea, and heat stress, with dire consequences for pregnant women and children (World Health Organization 2023).

Climate change poses a significant threat to the health of pregnant women and fetuses (Roos et al. 2021). Extensive research has shown that climate change, including extreme heat, wildfires, air pollution, floods, droughts, and food insecurity, can increase the risk of preterm birth, low birth weight, stillbirth (Chersich et al. 2020), congenital anomalies (Haghighi et al. 2021), pre‐eclampsia (Shashar et al. 2020), gestational diabetes (Pace, Vassallo, and Calleja‐Agius 2021), anemia (United States Environmental Protection Agency 2023), hypertensive disorders (Pedersen et al. 2017), placenta previa (Eltelt, Shafik, and Mohamed 2023) and respiratory diseases (Thangavel, Park, and Lee 2022). In addition, extreme weather and natural disasters have been associated with mental health outcomes, including depression, anxiety, and post‐traumatic stress disorder (Smith et al. 2023).

Health professionals play a crucial role in informing the public about the impact of climate change on health (Kircher et al. 2022). As the effects of climate change become more evident, it is critical to communicate these health risks effectively to the general public. Surveys have revealed that a significant majority of healthcare providers in the United States agree that climate change is occurring and that it directly or indirectly affects the health of their patients and communities (Boland and Temte 2019; Sarfaty et al. 2015, 2016). A recent exploratory review indicated that the role of healthcare professionals, individually or collectively, through professional organizations, could include informing and alerting patients, individuals, communities, and decision‐makers about the association of climate and environmental change with health and the need to act to limit and mitigate these risks to protect health. Various channels can be used, such as websites of healthcare organizations, interventions targeting decision‐makers, and highly visible reports (Dupraz and Burnand 2021). Healthcare professionals have a professional public health duty to foster the assessment and implementation of effective interventions, to improve the education of their peers, and to keep informing and alerting various audiences through potentially appropriate communication interventions (Barteit et al. 2019).

International organizations have established goals to combat climate change and mitigate its effects. The International Federation of Gynecology and Obstetrics (International Federation of Gynecology and Obstetrics 2020) places global health as its top priority and urges the recognition of the climate crisis as a worldwide emergency. It advocates for healthcare providers to assume leadership roles in education, advocacy, and research to address the changing health outcomes and the necessary global awareness. In addition, as highly trusted professionals, nurses play a crucial role in educating vulnerable populations, such as pregnant women, about the risks of exposure to climate change during pregnancy and providing them with guidance on how to protect themselves and their developing fetuses (Gaudreau et al. 2024).

Existing literature on the consequences and risks of climate change on maternal and fetal health is limited (Roos et al. 2021). Therefore, this study aimed to investigate the influence of climate change education on climate change awareness and anxiety levels in pregnant women.

## 2 Methods

### 2.1 Design and Ethical Principles of the Study

This study is a quasi‐experimental pre‐test (pre‐intervention) and a post‐test (post‐intervention) design. This study was conducted at a tertiary maternity hospital in northwestern Turkey, Istanbul. Zeynep Kamil Women and Children's Disease Hospital is one of Turkey's largest perinatal centers, serving patients from urban, suburban, and rural areas, and provides a diverse mix of study participants. This study included 1126 pregnant women attending the antenatal clinics of the hospital between April and June 2023. We assessed the climate change awareness, perception, knowledge, behavioral and policy expectations, and anxiety of 1126 pregnant women before and after the climate change education. This study followed the ethical principles outlined in the Declaration of Helsinki and was approved by the Research Ethics Committee (Approval: 47, 5.04.2023).

### 2.2 Sample Selection and Data Collection

The study participants were recruited through convenience sampling from the outpatient clinic waiting rooms of the hospital. Eligible participants were pregnant women between the ages of 18 and 45 years, with a gestational age greater than 28 weeks, who could speak, read, and write in Turkish. Exclusions were made for individuals under 18 or over 45 years of age, those with major depression or psychiatric illness, and those who refused to participate in the study (Table 1).

**TABLE 1:** Baseline characteristics of study participants (*n* = 1126).

	*n*, (%)
Educational level	
Primary School	101 (9.8)
Secondary School	317 (30.9)
High School	315 (30.7)
University	293 (28.6)
Occupation	
Government employee	111 (10.8)
Private employee	55 (5.4)
Daily laborer	23 (2.2)
Unemployed	837 (81.6)
Income	
Income is less than expense	16 (1.6)
Income is equal to expense	1010 (98.4)
Comorbidities	
No	969 (94.4)
Hypothyroidism	24 (2.3)
Diabetes mellitus	14 (1.4)
Hypertension	19 (1.9)
	Mean ± SD
Age (year)	28.44 ± 5.63
Gravida	2.11 ± 1.34
Gestational week	27.37 ± 9.75

*Note*: Data are shown as Mean ± Standard Deviation and number (*n*) and percentages (%).

Data were collected through face‐to‐face interviews with a trained midwife. The principal investigator followed the data collection procedure plan daily. The data collector received a 3‐day training program on data collection, sample selection, study tool administration, and data handling procedures. The data collector was familiar with the study's objectives, methods, and ethical aspects.

### 2.3 First Phase: Pre‐Test of the Study

Participants in the study were invited to participate in a survey on climate change. The purpose and design of the study were explained, and pregnant women were assured of voluntary participation, data confidentiality, and the right to withdraw at any time. Informed consent was obtained before data collection. Participants who expressed interest in participating answered a set of questions related to sociodemographics and completed the Climate Change Awareness Scale and the Climate Change Worry Scale.

### 2.4 Second Phase: Post‐Test of the Study

After completing the pre‐test, pregnant women were exposed to a brochure titled “Pregnancy and Climate Change,” published by the Health and Environment Alliance (HEAL), the FIGO, and the University of California, San Francisco (UCSF). The brochure provides insight into how climate change affects pregnancy and what women can do to address these effects. It presents a comprehensive overview of how climate change may increase health risks to expectant mothers and their children (Health and Environment Alliance 2024). The post‐test assessment was conducted after a 10‐min gap. Subsequently, the respondents were requested to retake both the Climate Change Awareness Scale and the Climate Change Worry Scale. The interview was designed to take approximately 30 min. A copy of the brochure distributed to the participants is included in the supporting information of the article (Supporting Appendix S1).

### 2.5 Participant

**FIGURE 1:**
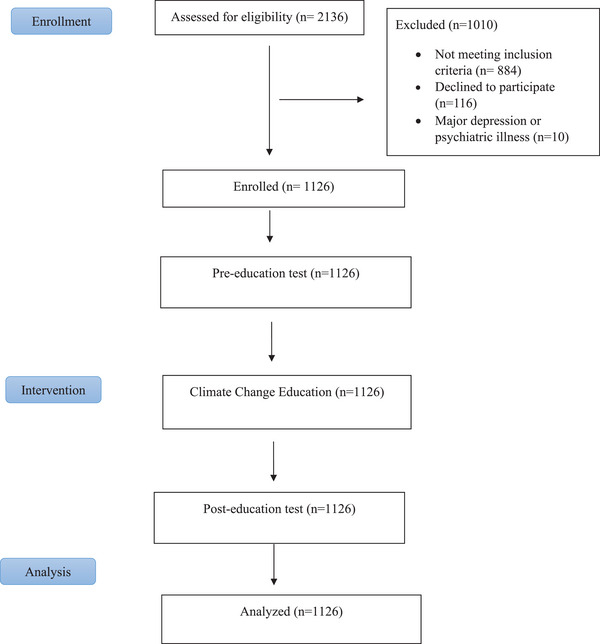
Flow diagram. [Colour figure can be viewed at wileyonlinelibrary.com]

A total of 2136 patients were assessed for eligibility, of which 1126 patients were included in the analysis. Of those, 1010 patients were excluded due to failure to meet the inclusion criteria (*n* = 884), the presence of major depression or psychiatric illness (*n* = 10), or refusal to participate (*n* = 116). The 1126 enrolled patients initially underwent a pre‐education test, received climate change education, and then completed a post‐education test. No patients were lost to follow‐up (Figure 1).

### 2.6 Defining the Instruments and Measurement

Climate Anxiety Scale, developed by Stewart (2021), was adapted to the Turkish by Gezer and İlhan (2021). The scale demonstrated a two‐factor structure, consisting of anxiety and feelings of helplessness. It is a 10‐item, 5‐point Likert scale. Cronbach's alpha coefficients for anxiety and helplessness were 0.87 and 0.83, respectively.

The Climate Change Awareness Scale was developed by Ataklı and Kuran (2022). It consisted of five factors and 52 items on a 5‐point Likert scale (strongly disagree (1) to strongly agree (5)). A total score is calculated by summing up the scores of all items, with higher scores indicating higher levels of awareness of climate change. These factors are climate change awareness, perception of the problem, knowledge of the causes of climate change, climate change anxiety, and expectations from behaviors and policies. The Cronbach's alpha of the total scale was 0.92; it was 0.93 for ‘‘the expectations from behaviors and policies’’, 0.91 for the ‘‘climate change anxiety’’, 0.87 for the ‘‘knowledge of the causes of climate change’’, 0.80 for the ‘‘climate change awareness’’, 0.81 for the ‘‘perception of the problem’’.

### 2.7 Statistical Analysis

The statistical analysis was conducted using IBM SPSS 25 software. Categorical variables were presented as frequency and percentage, while quantitative variables were described as the arithmetic mean and standard deviation. Pre‐ and post‐education measurements were compared using a paired sample *t*‐test, with statistical significance defined as a *p* value of less than 0.05.

## 3 Results

The study included responses from 1126 pregnant women with a mean age of 28.44 ± 5.63 years (range:18–44) and a mean gestational age of 27.37 ± 9.75 weeks (range: 10–37). The mean body mass index (BMI) was 28.3 ± 4.7 kg/m^2^ (range:17–40). Of the participants, 837 (81.6%) were unemployed, 317 (30.9%) graduated from secondary school, 969 (94.4%) had no comorbidities, and 1007 (98.1%) stated that their monthly income was equal to expenses.

To investigate the impact of climate change education, we compared the pre‐ and post‐questionnaire datasets. The findings indicated a statistically significant reduction in anxiety levels regarding climate change among pregnant women following climate change education (*t* = 28.958, *p* < 0.001). Furthermore, the data indicated a significant increase in feelings of helplessness among pregnant women following climate change education (*t* = 15.655, *p* < 0.001).

The findings revealed a significant increase in the awareness (*t* = −75.852, *p* < 0.001) and perception of pregnant women (*t* = −46.705, *p* < 0.001) following climate change education. Similarly, there was a significant improvement in the knowledge of pregnant women after climate change education (*t* = ‐42.908, *p* < 0.001). Furthermore, a significant enhancement was noted in behavioral and policy expectations of pregnant women regarding climate change following climate change education (*t* = −58.015, *p* < 0.001). A significant decline in anxiety levels was observed in pregnant women following climate change education (*t* = 54.148, *p* < 0.001) (Table 2).

**TABLE 2:** Comparison of scale scores before and after climate change education.

	Before climate change education	After climate change education	*t*	*p* value
Climate change worry scale				
Anxiety	15 ± 6.06	12.38 ± 3.62	28.958	< 0.001^*^
Feeling of helplessness	4.3 ± 2.03	12.38 ± 1.27	15.655	< 0.001^*^
Total	19.3 ± 7.42	16.26 ± 4.46	28.45	< 0.001^*^
Climate change awareness scale				
Awareness	22.82 ± 8.61	29.59 ± 6.04	−75.852	< 0.001^*^
Perception	12.38 ± 6.34	15.48 ± 4.54	−46.705	< 0.001^*^
Knowledge	24.85 ± 10.65	29.46 ± 7.62	−42.908	< 0.001^*^
Anxiety	32.29 ± 11.41	25.6 ± 8.34	54.148	< 0.001^*^
Behavioral and policy expectations	51.37 ± 19.41	63.93 ± 13.38	−58.015	< 0.001^*^
Total	143.71 ± 52.82	164.06 ± 37.15	−39.346	< 0.001^*^

*Note*: Data are shown as Mean ± Standard Deviation and number (*n*) and percentages (%).

* *p* < 0.05 was considered statistically significant.

## 4 Discussion

The study suggests that climate change education may reduce climate change anxiety among pregnant women and improve their awareness, perceptions, knowledge, behaviors, and policy expectations about climate change. In addition, the study found that climate change education was associated with an increase in feelings of helplessness among pregnant women.

The existing literature has yet to establish the definitive impact of information exposure on climate change attitudes. The influence of information exposure on attitudes towards climate change remains a topic of debate, with conflicting results from various studies. A study in Bolivia found no significant effect of information exposure on perceptions of climate change (Fernández‐Llamazares et al. 2015). Conversely, an Indian study suggested that exposure to climate change‐related television media increased risk perceptions, while internet exposure had a negative effect, and newspaper exposure showed no significant impact (Thaker, Zhao, and Leiserowitz 2017). Meanwhile, a study in Japan demonstrated a positive correlation between exposure to climate change‐related media reports and the level of concern expressed by individuals (Sampei and Aoyagi‐Usui 2009). Additionally, an Italian study among university students showed that exposure to climate change information leads to climate anxiety and increased self‐efficacy (Maran and Begotti 2021).

Another study examining the predictors of climate anxiety and its relation to pro‐environmental behavior and mental well‐being found that exposure to information via the media about the impacts of climate change was significantly associated with climate anxiety. However, exposure to information about possible solutions to climate change did not exhibit any association with climate anxiety (Ogunbode et al. 2022). The findings of our study showed that the exposure of pregnant women to climate change education by healthcare providers was negatively associated with climate change anxiety and led to an increase in awareness, perception, knowledge, behavioral changes, and policy expectations related to climate change. The significant impact of the information delivered by healthcare providers on climate change anxiety, awareness, perception, knowledge, behavioral changes, and policy expectations represents a crucial avenue for further research.

Healthcare professionals play a pivotal role in educating the public regarding the health implications of climate change. As the effects of climate change become increasingly evident, effective communication of health risks is crucial. Surveys indicate that most US healthcare providers recognize climate change and its direct or indirect effects on patient and community health (Sarfaty et al. 2016a). A recent exploratory review underscores the multifaceted role of healthcare professionals in addressing climate‐related health risks, encompassing patient education, community outreach, engagement with decision‐makers, and advocacy for preventive measures (Dupraz and Burnand 2021). Diverse communication channels, including healthcare organization websites, can facilitate these efforts. It is incumbent upon healthcare professionals to implement effective interventions, enhance peer education, and engage with diverse audiences through appropriate communication strategies to mitigate climate‐related health risks (Barteit et al. 2019).

It is noteworthy that the educational interventions were relatively brief. The average time spent by pregnant women engaged in the study was 30 min. This included a review of the educational content and responses to all the evaluation queries. In contexts where attention and time are limited, it is crucial to use engaging materials, such as brochures, to maximize the time and attention devoted to the message. This helps to enhance the generalizability of our findings, given the widespread use of short‐format patient education materials in healthcare settings.

Climate change education enables individuals to understand and address the impacts of the climate crisis, empowering them with the necessary knowledge, skills, values, and attitudes to act as agents of change (United Nations Educational, Scientific, and Cultural Organization 2023). Healthcare professionals are considered a reliable source of information on climate change (Maibach, Frumkin, and Ahdoot 2021). As understanding the impacts of climate change on public health deepens, it is crucial to implement measures to protect individuals from its detrimental effects (Watts et al. 2019). A study comparing the effectiveness of narrative versus traditional didactic information on pregnant women's perceptions of climate change demonstrated the benefits of a narrative approach in educating pregnant women about the maternal and child health threats posed by climate change (Adebayo et al. 2020). Our findings suggest that climate change education provided by health professionals can alleviate climate change anxiety among pregnant women while enhancing their awareness, perceptions, knowledge, behaviors, and policy expectations.

The awareness and perception of current and future climate‐related threats, coupled with insufficient climate action, have been associated with a range of psychological and emotional responses, including anxiety, stress, distress, hopelessness, fear, anger, grief, helplessness, frustration, and guilt. Recent research suggested that individuals with increased awareness and knowledge about climate change are more likely to experience higher levels of climate change anxiety and hopelessness. In addition, a study focusing on young people revealed that, despite their awareness of climate change and its impacts, their concerns are not adequately acknowledged, leading to feelings of hopelessness and powerlessness. The emotional toll may encompass anxiety, depression, anger, sadness, frustration, grief, and powerlessness. As knowledge about the climate crisis increases, it becomes apparent that the more people feel helpless in their ability to take action, the more they experience a sense of hopelessness. Our findings align with prior research. Future investigations need to explore potential interventions, policies, and decision‐making processes aimed at alleviating the mental health impacts of climate change while also producing robust evidence on the association between mental health and climate change.

### 4.1 Limitations

The present study is subject to several limitations. Our study used opportunity sampling and a participant pool from a tertiary referral maternity hospital. As a result, Our findings may not be representative of all pregnant women living in other parts of the world. It is important to acknowledge that the manifestations of climate anxiety may vary across different demographic subgroups that were either overrepresented or underrepresented in the study. Therefore, further studies with representative national samples are necessary to fully understand the generalizability of our findings. Another limitation is the absence of a control condition and lack of a follow‐up measure to ascertain if the observed improvements were retained over time. The data were collected between April and June 2023. The seasonal variability may represent a limiting factor in this study. Despite these limitations, there are several strengths of this study. Data were collected using validated scales. To date, numerous studies have underscored the necessity of identifying research‐based strategies to alleviate the impacts of climate change on the health of populations such as university students and young adults. Our study may be unique in its quest to provide a basis for determining the effects of climate change education on pregnant women's anxiety and awareness.

## 5 Conclusions

The findings of this study suggest that climate change education may alleviate climate change anxiety among pregnant women while enhancing their awareness and improving their perceptions, knowledge, behaviors, and policy expectations about climate change.

## Ethics Statement

Written informed consent was sought from each pregnant woman following an explanation of the purpose and design of the study. Pregnant women were informed that their involvement in the study was voluntary and that they could withdraw at any time. In addition, they were given the assurance that their data would be kept confidential. This study followed the ethical principles outlined in the Declaration of Helsinki and was approved by the Research Ethics Committee (Approval: 47, 5.04.2023).

## Conflicts of Interest

The authors declare no conflicts of interest.

## Supporting information



Supporting information

## Data Availability

The data that support the findings of this study are available on reasonable request from the corresponding author.
