# Supplementary material for: Impact of Climate Change Education on Pregnant Women's Anxiety and Awareness
Source: Public Health Nurs. 2024 Oct 10;42(1):54–60. doi: 10.1111/phn.13455 (PMC11700939; doi:10.1111/phn.13455)
Supplement: Supplementary file 1 — Supporting information [file PHN-42-54-s001.pdf]

How Does Climate Change and Air Pollution Affect Pregnancy and Human Development?

Climate change puts pregnant women and children at risk

The impacts of climate change are already taking place: more frequent natural disasters, extreme weather and temperatures, rising sea levels, and displacement. Mainly because of combustion of fossil fuels, CO_2 is causing warming, and these changes affect food and housing security, vector-borne illness, and access to clean air and water, all of which influence human health, especially that of pregnant women and children. Research shows evidence linking climate change with poor pregnancy outcomes that can have lasting effects on children and the health of subsequent generations [1].

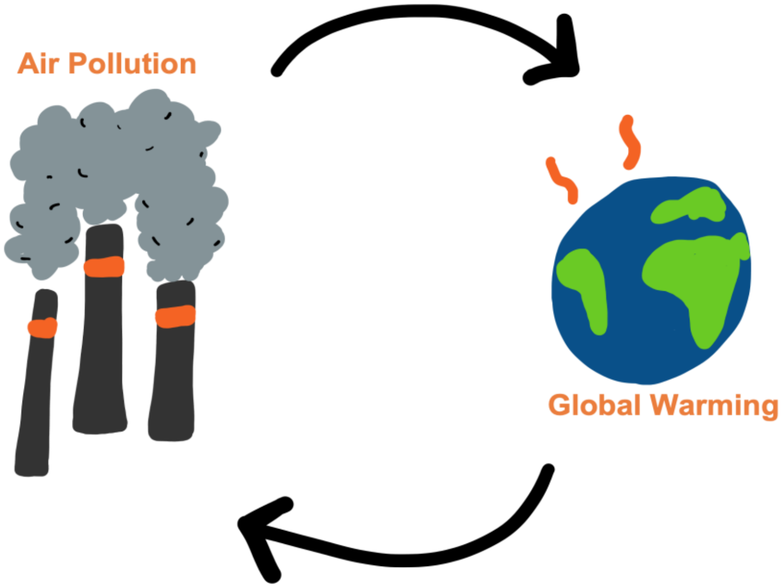

Warming is making air pollution worse

Global warming makes air pollution worse by extending the ozone season and leading to droughts and storms that contribute to forest fires and widespread smoke. Many of the same sources of air pollution ($PM_{2.5}$, VOCs, NO_x) also emit carbon dioxide (CO_2), the main culprit causing global warming [2]. These sources include burning fossil fuels (e.g. coal-fired power plants, gasoline-powered cars and diesel trucks). Reducing CO_2 emissions can reduce these types of air pollution and the health damages that result [3].

Air pollution increases risks to reproductive health and human development

Physiological changes during pregnancy, such as a 40% increase in the amount of air pregnant women breathe per minute as well as a 50% increase in how hard their hearts, work make pregnant women particularly vulnerable to pollutants [4,5,6,7].

Research shows that prenatal exposure to air pollutants $PM_{2.5}$ and ozone contribute to adverse pregnancy outcomes including preterm birth [8,9,10,11] and low birthweight [9,10,11]. Meta-analyses have found that over 3% of preterm birth in the United States and 18% of preterm birth globally could be attributed to $PM_{2.5}$ exposure [12,13].

Heat and the risk to reproductive health and human development

Global warming increases extreme heat which is associated with preterm birth, low birthweight, and stillbirth in studies across the world [14-23]. Increasing temperatures and number of hot days are associated with low birth weight [23,24].

What can we do?

Advocating for public policies that promote clean air and prevent climate change is essential. When counseling patients on mitigating risk of exposure to air pollution and heat, consider the following:

1. Avoid outdoor activities on high pollution days and monitor the Air Quality Index in your area.
2. Close your windows on high pollution days.
3. When exercising, avoid being within 300 meters of high-traffic areas.
4. During extreme heat, limit outdoor activities to morning or evening.
5. Increase fluid intake on hot days as doing so has been associated with lower risk for heatstroke.
6. Avoid being in sun for long periods and seek shade, take breaks and drink water regularly.
7. Access air conditioning if possible to reduce risk for heatstroke or heat-related mortality. [25-29]

We can all do our part to reduce air pollution and prevent the climate crisis. This includes walking, biking, carpooling and using public transportation when possible. Conserve energy by supporting renewable energy (solar, wind) and shutting off lights when not in use at home and at work and buy energy efficient appliances where available. Don't use aerosol sprays; instead, use cleaning products and paints that are environmentally safe.

Protective public policies are essential for health

Health care providers are a trusted voice for primary prevention at the policy level. Health care providers are especially effective at advocating for public policies that protect patients and communities by reducing pollutants and global warming and building resilience in the health care sector [30,31,32].

Policy interventions that reduce prenatal exposure to particulate matter, global reduction in CO_2 emissions, and divestment from fossil fuels can help to ensure the health of everyone, especially pregnant people and children.

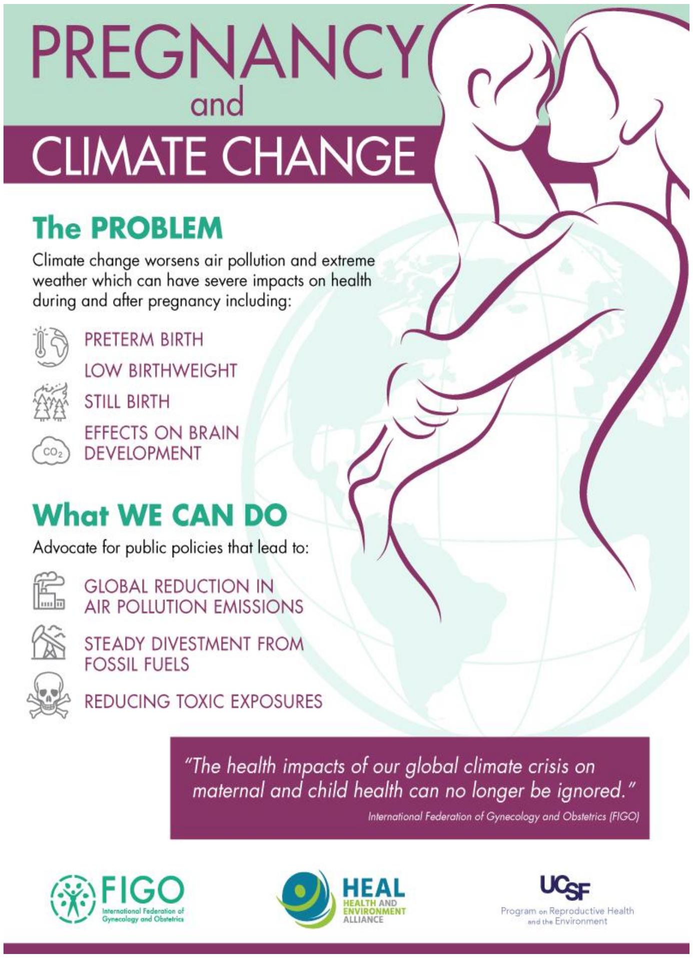

Kiana Nguyen, medical student UCSF, Annemarie Charlesworth, MA, UCSF, Marya Zlatnik, MD, UCSF, Tracey J. Woodruff, PhD, Professor and Director, UCSF Program on Reproductive Health and the Environment, Génon K. Jensen, Executive Director, Health and Environment Alliance (HEAL), Vlatka Matkovic Puljic, PhD, Senior Policy Officer, Health and Energy Program, Health and Environment Alliance (HEAL), and Patricia D. Koman, MPP, PhD, President, Green Barn Research

References available at prhe.ucsf.ed

References for Climate Change and Pregnancy Fact Sheet

1. Watts N, Amann M, Arnell N, et al. The 2019 report of The Lancet Countdown on health and climate change: ensuring that the health of a child born today is not defined by a changing climate. Lancet. 2019 Nov 16;394(10211):1836-1878.
2. USGCRP, 2018: Impacts, Risks, and Adaptation in the United States: Fourth National Climate Assessment, Volume II [Reidmiller, D.R., C.W. Avery, D.R. Easterling, K.E. Kunkel, K.L.M. Lewis, T.K. Maycock, and B.C. Stewart (eds.)]. U.S. Global Change Research Program, Washington, DC, USA, 1515 pp. doi: 10.7930/NCA4.2018.
3. https://health2016.globalchange.gov/
4. Dreiling CE, Carman FS III, Brown DE. Maternal endocrine and fetal metabolic responses to heat stress. J Dairy Sci.1991;74:312–327.
5. Elkus R, Popovich J. Respiratory physiology in pregnancy. Clin Chest Med. 1992;13(4):555.
6. Prowse CM, Gaensler EA. Respiratory and acid-base changes during pregnancy. Anesthesiology. 1965;26:381.
7. Koman PD, Hogan KA, Sampson N, et al. Examining Joint Effects of Air Pollution Exposure and Social Determinants of Health in Defining "At-Risk" Populations Under the Clean Air Act: Susceptibility of Pregnant Women to Hypertensive Disorders of Pregnancy. World Med Health Policy. 2018 Mar;10(1):7-54.
8. DeFranco E, Moravec W, Xu F et al. Exposure to airborne particulate matter during pregnancy is associated with preterm birth: a population-based cohort study. Environ Health. 2016;15:6.
9. Ha S, Hu H, Roussos-Ross D, Haidong K, Roth J, Xu X. The effects of air pollution on adverse birth outcomes. Environ Res. 2014 Oct;134:198-204.
10. Lamichhane DK, Leem JH, Lee JY, Kim HC. A meta-analysis of exposure to particulate matter and adverse birth outcomes. Environ Health Toxicol. 2015 Nov 3;30:e2015011.
11. Li X, Huang S, Jiao A et al. Association between ambient fine particulate matter and preterm birth or term low birth weight: An updated systematic review and meta-analysis. Environ Pollut. 2017 Aug;227:596-605.
12. Trasande L, Malecha P, Attina TM. Particulate Matter Exposure and Preterm Birth: Estimates of U.S. Attributable Burden and Economic Costs. Environmental Health Perspectives 2016;124:12.
13. Malley CS, Kuylenstierna JC, Vallack HW, et al. Preterm birth associated with maternal fine particulate matter exposure: A global, regional and national assessment. Environment International. Environ Int. 2017 Apr;101:173-182.
14. Basu R, Sarovar V, Malig BJ. Association Between Ambient Temperature and Risk of Stillbirth in California. Am J Epidemiol. 2016 May 15;183(10):894-901.
15. Bruckner TA, Modin B, Vagero D. Cold ambient temperature in utero and birth outcomes in Uppsala, Sweden, 1915-1929. Ann Epidemiol. 2014 Feb;24(2):116:21.
16. Carolan-Olah M, Frankowska D. High environmental temperature and preterm birth: a review of the evidence. Midwifery. 2014 Jan;30(1):50-9.
17. Ha S, Liu D, Zhu Y, et al. Ambient temperature and early delivery of singleton pregnancies. Environ Health Perspect. 2017 Mar;125(3):453-459.
18. Kuehn L, McCormick S. Heat Exposure and Maternal Health in the Face of Climate Change. Int J Environ Res Public Health. 2017 Jul;14(8).
19. Poursafa P, Keikha M, Kelishadi R. Systematic review on adverse birth outcomes of climate change. J Res Med Sci. 2015 Apr;20(4):397-402.
20. Rylander C, Odland JØ, Sandanger TM. Climate change and the potential effects on maternal and pregnancy outcomes: an assessment of the most vulnerable--the mother, fetus, and newborn child. Glob Health Action. 2013 Mar 11(6), 19538.
21. Strand LB, Barnett AG, Tong S. Maternal exposure to ambient temperature and the risks of preterm birth and stillbirth in Brisbane, Australia. Am J Epidemiol. 2012 Jan 15;175(2):99-107.
22. Zhang Y, Yu C, Wang L. Temperature exposure during pregnancy and birth outcomes: An updated systematic review of epidemiological evidence. *Environ Pollut*. 2017 Jun; 225:700-712.
23. Grace K, Davenport F, Hanson H, et al. Linking climate change and health outcomes: examining the relationship between temperature, precipitation and birth weight in Africa. Global Environ Chang. 2015 Nov; 35:125-137.
24. Kloog I, Melly SJ, Coull BA, et al. Using satellite-based spatiotemporal resolved air temperature exposure to study the association between ambient air temperature and birth outcomes in Massachusetts. Environ Health Perspect. 2015 Oct; 123(10):1053-1058.
25. Kilbourne EM, Choi K, Jones TS, Thacker SB. Risk factors for heatstroke: A case-control study. JAMA. 1982 Jun 25; 247(24):3332-6.

26. Semenza JC, Rubin CH, Falter KH, et al. Heat-related deaths during the July 1995 heat wave in Chicago. N Engl J Med. 1996 Jul 11; 335(2):84-90.
27. Laumbach R, Meng Q, Kipen H. What can individuals do to reduce personal health risks from air pollution? J Thorac Dis. 2015 Jan;7(1):96-107.
28. Lin LY, Chuang HC, Liu IJ. Reducing indoor air pollution by air conditioning is associated with improvements in cardiovascular health among the general population. Sci Total Environ. 2013 Oct 1;463-464:176-81.
29. https://www.epa.gov/sites/production/files/2016-10/documents/extreme-heat-guidebook.pdf
30. https://www.apha.org/-/media/files/pdf/topics/climate/health_and_climate_resource_guide.ashx
31. https://climateforhealth.org/cfh-moving-forward-guide/
32. https://toolkit.climate.gov/topics/human-health/building-climate-resilience-health-sector
